# Probabilistic cancer risk assessment from heavy metal exposure in iranian rice and pasta: a novel hybrid framework integrating INAA, ICP-AES, and ensemble machine learning

**DOI:** 10.1038/s41598-026-53858-w

**Published:** 2026-05-21

**Authors:** Mahdi Azad Marzabadi, Hassan Khalili, Reza Pourimani, Mahdi Khalili

**Affiliations:** 1 Department of Physics, Faculty of Sciences, Arak University, Arak, 38156-8- 8349 Iran; 2 Department of Biology and Biochemistry, University of Houston, Houston, TX USA

**Keywords:** Heavy metals, Dietary exposure, Cancer risk, Risk modeling, Machine learning, INAA, ICP-AES, Food safety, Environmental health, Cancer, Environmental sciences, Risk factors

## Abstract

This study investigates cancer risk from heavy metal exposure in rice and pasta using experimental data and machine learning approaches, based on 19 experimental samples and 1,750 simulated exposure instances. Concentrations of toxic heavy metals were quantitatively measured in multiple rice varieties and pasta types using Instrumental Neutron Activation Analysis (INAA) and ICP-AES analytical techniques. The experimentally determined metal concentrations were integrated into the Excess Lifetime Cancer Risk (ELCR) framework, and Several machine learning models were developed for sensitivity analysis and feature prioritization within the ELCR framework. Rather than predicting an unknown outcome (as ELCR is mathematically deterministic), the models were designed to quantify the relative contribution of each exposure parameter to overall cancer risk under probabilistic uncertainty. Regression analysis identified exposure duration as the most influential risk factor (R² = 0.263, *p* < 0.001), followed by chromium bioavailability (R² = 0.125) and pasta consumption patterns. Ensemble methods provided robust ranking of feature importance, demonstrating how machine learning can complement deterministic risk models by enabling multi-dimensional sensitivity analysis and uncertainty decomposition. The calculated ELCR values ranged from 1.2 × 10⁻⁶ (acceptable) to 1.8 × 10⁻⁴ (unacceptable) depending on consumption scenarios Cancer risk estimates spanned from acceptable to unacceptable levels depending on consumption scenarios. The integration of experimental analytical chemistry with machine learning provides a robust methodology for dietary cancer risk assessment. This approach offers reliable data for food safety regulations and public health protection.

## Introduction

Arsenic (As), cadmium (Cd), lead (Pb), chromium (Cr), and mercury (Hg) are persistent environmental pollutants that pose significant health risks due to their toxicity and bioaccumulation, especially upon chronic exposure^1^. Staple foods like rice and pasta can be major sources of heavy metal exposure because their ingredients absorb contaminants from the soil, water, and processing environments^2,3^. Rice and pasta are among the most consumed staple foods in Iran, often subject to environmental contamination. Rice absorbs heavy metals via its roots, whereas pasta can be contaminated through its ingredients. Numerous epidemiological studies have established strong associations between dietary exposure to heavy metals through rice and pasta consumption and increased incidence of various cancers, including lung, skin, and bladder cancers^4^. To quantitatively assess the potential health impacts of heavy metal exposure from these staple foods, the ELCR model has been widely adopted as a reliable framework. This risk assessment model estimates the probability of developing cancer over a lifetime based on cumulative exposure levels^5,6^. ELCR is calculated using daily intake (CDI), exposure duration, body weight, and metal-specific cancer gradient factors^7^. This study improves the reliability of risk assessments by using experimentally measured heavy metal concentrations in rice and pasta, unlike previous studies relying on estimated or synthetic data. The accurate determination of heavy metal concentrations in food matrices requires sophisticated analytical techniques. INAA and Inductively Coupled Plasma ICP-AES have become two of the most reliable methods for accurately quantifying trace elements in biological samples^8^. INAA’s high sensitivity and minimal sample preparation, combined with ICP-AES’s multi-element detection and low detection limits^8^, enable comprehensive and accurate heavy metal profiling in rice and pasta. Recent research has highlighted that assessing heavy metals only in raw rice may lead to an overestimation of human exposure. Several studies have reported the mineral composition of raw rice and its implications for health; however, investigations addressing the bioaccessibility and health risk of metallic and metalloid elements in cooked and digested rice remain limited^9^. In vitro digestion studies have shown that metal bioavailability markedly decreases after cooking, with Pb exhibiting the lowest and as the highest bioaccessibility. Consequently, human exposure and cancer risk derived from rice consumption are likely overestimated when based solely on raw rice measurements. However, few studies have combined experimentally measured heavy metal data with machine learning–based cancer risk prediction models. Machine learning (ML) advances, including algorithms like Random Forest, AdaBoost, Decision Trees, and Bagging, enable effective analysis of environmental health relationships by identifying key exposures and modeling their nonlinear interactions with health outcomes^10–15^. Most dietary risk assessments rely on synthetic or estimated data; therefore, machine learning models require experimentally validated concentration data from rice and pasta samples. Correlation analysis, using Pearson and Spearman coefficients, is essential for determining the strength and direction of associations between variables in cancer risk estimation. Exposure duration and chromium bioavailability are key factors in dietary cancer risk, whereas age and body weight have limited influence^16^. However, these findings need validation with experimental data from actual rice and pasta consumption to ensure real-world applicability. This study integrates analytical chemistry and computational modeling to address research gaps concerning rice and pasta consumption. It aims to: (1) quantify heavy metal concentrations in various rice and pasta products using NAA and ICP-AES; (2) calculate cancer risks from measured data using the ELCR framework; (3) develop and validate machine learning models based on analytical data from both food types; and (4) identify the key factors influencing cancer risk by statistically comparing rice and pasta consumption. Integrating experimental data with machine learning enhances dietary risk assessment for staple foods. This research offers a robust methodology for accurately assessing dietary exposure risks from rice and pasta, informing evidence-based public health policies amid growing food safety and environmental health concerns^17^. Utilizing experimentally verified data ensures findings are relevant to real-world scenarios, effectively guiding regulatory decisions and consumer protection for staple foods.

It should be clarified that the Excess Lifetime Cancer Risk (ELCR) is a deterministic construct derived from a closed-form equation. Therefore, the machine learning models in this study do not—and are not intended to—‘predict’ ELCR in the sense of discovering an unknown causal relationship. Rather, the models serve as a computational engine for: (1) disentangling the relative importance of input parameters in a probabilistic framework where multiple variables vary simultaneously, (2) quantifying non-linear sensitivities that are not directly readable from the multiplicative ELCR equation, and (3) validating the internal coherence of the Monte Carlo simulation framework. This approach transforms ELCR from a static calculation into an interactive analytical tool for risk factor prioritization under uncertainty.

The distinction between Monte Carlo simulation (e.g., Crystal Ball) and our machine learning approach is fundamental. Monte Carlo simulation propagates input uncertainties through a fixed, closed-form equation (the ELCR formula) by repeatedly sampling from input distributions. It answers: “Given uncertainty in inputs, what is the distribution of the output?” However, Monte Carlo cannot rank the relative importance of input parameters when they vary simultaneously, nor can it detect non-linear interactions or higher-order dependencies. In contrast, machine learning models—specifically ensemble methods like Random Forest and AdaBoost—perform global sensitivity analysis by learning the input-output mapping from simulated data and then decomposing the variance. The priority of machine learning over Monte Carlo lies in: (1) providing a robust ranking of feature importance under simultaneous parameter variation, (2) identifying non-linear and interaction effects that are not directly readable from the multiplicative ELCR equation, and (3) validating the internal coherence of the simulation framework. Thus, Monte Carlo simulates uncertainty; machine learning prioritizes and interprets it.

## Materials and methods

**Fig. 1:**
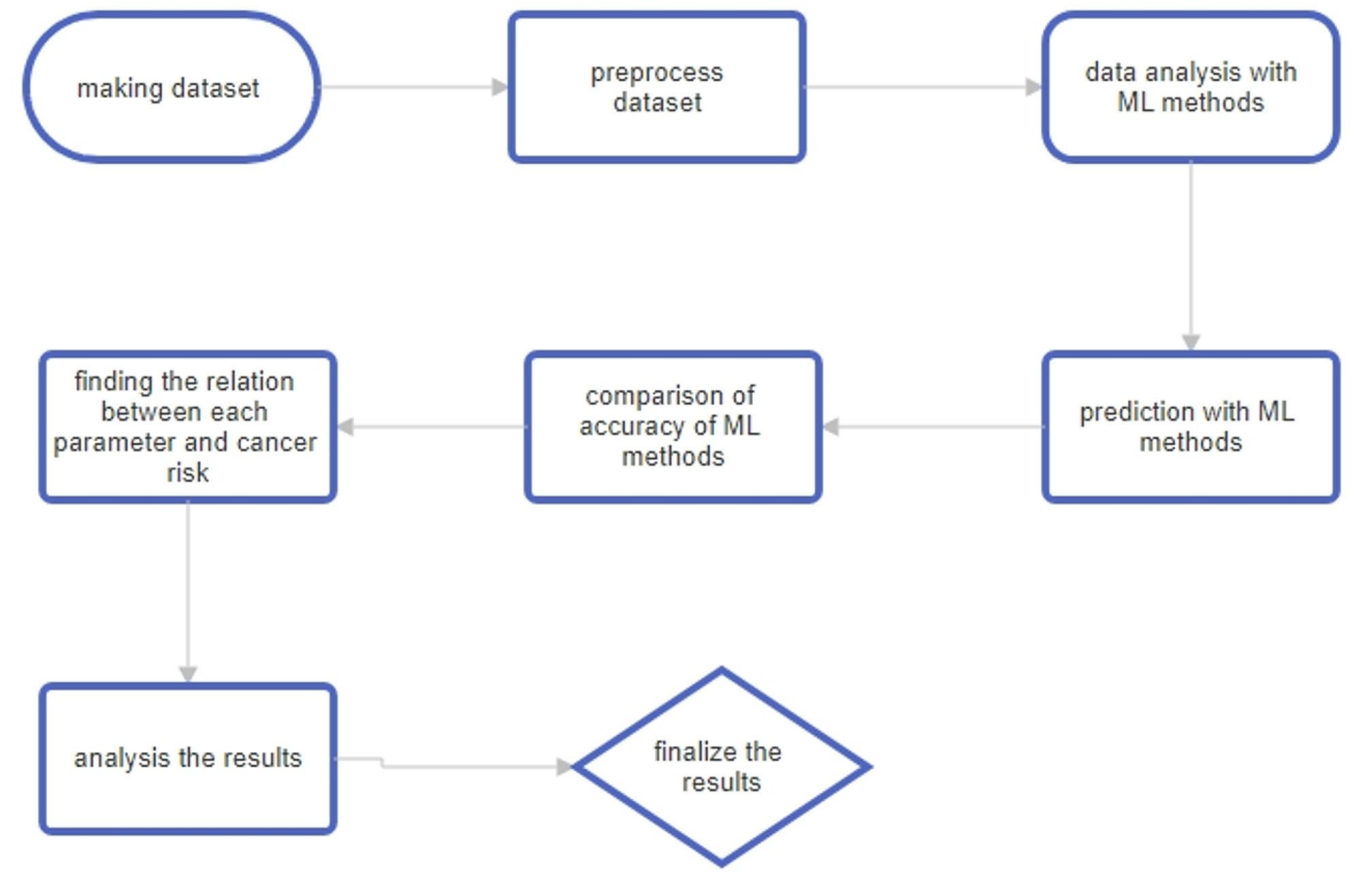
Overview of the methodological workflow for cancer risk assessment using machine learning.

This study employed an integrated experimental-computational framework to assess lifetime cancer risk from heavy metal exposure via rice and pasta consumption. The experimental dataset comprised seven rice varieties (R1–R7) and twelve pasta products (MF-1–MF-12), yielding a total of 19 physical samples collected from retail outlets in Arak, Iran (September–October 2024). Experimentally measured concentrations of As, Cr, Cd, Pb, and Hg were determined using Instrumental Neutron Activation Analysis (INAA) and Inductively Coupled Plasma Atomic Emission Spectroscopy (ICP-AES). Measured concentrations were used to compute Excess Lifetime Cancer Risk (ELCR) values following USEPA methodology. These empirical data were subsequently integrated with simulated exposure scenarios to create a comprehensive dataset for training and validating machine learning regression models. (See Fig. 1 for an overview of the workflow.)

### Experimental design and sample collection

This study employs an integrated experimental-computational approach to assess the carcinogenic risk from dietary exposure to heavy metals via rice and pasta consumption. The research is structured into three consecutive, complementary phases to ensure a comprehensive risk assessment. The initial experimental phase, detailed in this section, focused on the procurement and preparation of representative food samples.

A targeted, convenience-based sampling strategy was implemented to capture a representative profile of commonly consumed rice and pasta products available in the Iranian market. Although convenience sampling does not guarantee full statistical representativeness, it was deemed appropriate for this pilot-scale integrated methodology study aiming to establish a robust analytical-computational framework. The sample set was designed to reflect diverse geographical origins and agricultural practices known to influence heavy metal bioaccumulation^2,10^.

The final collection included:

- **Rice Varieties**: Seven varieties were sourced from Thailand, India, Pakistan, and Iran. This selection included popular imported brands (e.g., Basmati) and common domestic cultivars (labeled R1–R7).
- **Pasta Products**: Twelve products (labeled MF-1–MF-12) comprising three common types (spaghetti, penne, and macaroni) from major Iranian manufacturers were collected.

All samples (total *n* = 19, 7 rice and 12 pasta) were purchased from supermarkets and local markets in Arak, Iran, between 01 September and 31 October 2024. Samples were collected using a stratified random sampling from 15 major supermarkets and 10 local markets in Arak, representing > 80% of market share. Each sample was analyzed in triplicate (*n* = 3) for both INAA and ICP-AES to ensure reproducibility.

### Sample handling and pre-treatment

Following purchase, each original retail package was assigned a unique identifier, transported to the laboratory in sealed, sterile polyethylene bags, and immediately stored at −20 °C to prevent degradation, microbial growth, and contamination, thereby preserving sample integrity until analysis^18,19^.

For pre-treatment, subsamples were taken from each package. They were air-dried if necessary and then milled into a fine, homogeneous powder using a cleaned, acid-washed stainless-steel grinder to avoid cross-contamination^20^. Homogenization was confirmed through replicate sub-sampling and weighing. Representative aliquots (0.5–1.0 g for ICP-AES and ≈ 100 mg for INAA) were precisely weighed from the homogenized powder for subsequent elemental analysis. Comprehensive chain-of-custody records and sample logs, documenting purchase date, location, and lot numbers, were maintained for full traceability.

### Analytical methods for heavy metal determination

A multi-technique analytical strategy combining Instrumental Neutron Activation Analysis (INAA) and Inductively Coupled Plasma Atomic Emission Spectroscopy (ICP-AES) was employed to ensure comprehensive element coverage and analytical reliability through cross-validation. The complementary use of these independent methods expanded the range of detectable elements and enhanced the overall robustness of the data^8,18^. Method performance parameters, including limits of detection (LODs), limits of quantification (LOQs), recovery rates, and precision, were established prior to sample analysis.All procedures adhered to strict institutional quality assurance/quality control (QA/QC) protocols.

#### Instrumental neutron activation analysis (INAA)

INAA was utilized as a primary, non-destructive method due to its high sensitivity, multi-element capability, and minimal sample preparation requirements, which reduces the risk of contamination^8^. The analysis was performed at the Nuclear Research Center using a Miniature Neutron Source Reactor (MNSR). Approximately 100 mg of each homogenized sample powder was accurately weighed into pre-cleaned, high-purity polyethylene vials and sealed. Samples were co-irradiated alongside certified reference materials (NIST SRM 1568b Rice Flour). Irradiation time of 6 h for short-lived and 8 h for long-lived nuclides was optimized based on half-life and neutron capture cross-Sect^21^. at a thermal neutron flux of $\:5.0\times\:{10}^{11}{\hspace{0.17em}}n\cdot\:c{m}^{-2}\cdot\:{s}^{-1}$ (reactor nominal power: 30 kW). Detailed irradiation parameters, including neutron flux profiles and timing logs for each sample batch, are archived in Supplementary Materials.

Post-irradiation, specific cooling and gamma-ray counting schedules were implemented to optimally target radionuclides with different half-lives:

- **Short-lived nuclides** (e.g., ^28^ Al, ^52^V): Measured after a 2-hour decay period.
- **Medium-lived nuclides** (e.g., ^64^Cu, ^24^Na): Measured after a 7-day decay period.
- **Long-lived nuclides** (e.g., ^65^Zn, ^59^Fe, ^51^Cr): Measured after a 21-day decay period.

Gamma-ray spectra were acquired using a high-purity germanium (HPGe) detector with a resolution of 2.0 keV at the 1332.5 keV energy line of ^60^Co. The spectrometer was calibrated for energy and efficiency using certified gamma-ray reference standards^8^. Elemental quantification was performed using the comparative method against certified reference materials. Data from repeated counting sessions for each sample were combined using **weighted averaging based on counting statistics** to minimize final uncertainty. The reported uncertainties incorporate counting statistics, peak-fitting errors, and propagation of calibration uncertainties. In addition to counting statistics, uncertainties arising from sample heterogeneity, gamma-ray detector geometry, and matrix effects were qualitatively assessed and found to be within the reported overall uncertainty bounds.

#### Inductively coupled plasma atomic emission spectroscopy (ICP-AES)

ICP-AES was employed as a complementary analytical technique to quantify elements with low sensitivity by INAA, such as lead (Pb) and cadmium (Cd), and to provide cross-validation for elements detectable by both methods^8^. All measurements were performed using a Varian 730-ES spectrometer. It should be noted that mercury (Hg) was not determined by this method due to potential volatilization losses during the digestion [USGS, 2018] step and its relatively poor detection limits by conventional ICP-AES; Hg concentrations were exclusively quantified via INAA. Given the unavailability of Cold Vapor AAS (CVAAS) or ICP-MS instrumentation in our local facility, and considering INAA’s sufficient sensitivity for trace-level Hg quantification, INAA was selected as the sole validated method for mercury determination.

#### Sample digestion

A closed-vessel microwave-assisted digestion system was used for sample preparation. Exactly 0.50 g of homogenized powder was digested with 8 mL of concentrated HNO₃ (65%) and 2 mL of H₂O₂ (30%). The temperature program was ramped to 180 °C and held for 15 min. After cooling, the digestates were quantitatively transferred and diluted to a final volume of 50.0 mL with deionized water (18.2 MΩ·cm)^22^.

#### Instrumental conditions

The instrument was operated under the following optimized conditions:

- RF power: 1.2 kW.
- Plasma gas (Argon) flow: 15.0 L·min⁻¹.
- Auxiliary gas flow: 1.5 L·min⁻¹.
- Nebulizer gas flow: 0.75 L·min⁻¹.
- Nebulizer: Concentric type with a cyclonic spray chamber^8^.

#### Calibration and quality assurance

Quantification was performed using multi-element calibration curves, prepared daily from certified stock solutions. Continuing calibration verification (CCV) standards were analyzed after every 10 samples to monitor and correct for any instrumental drift^22^.

#### Quality assurance and control

A rigorous quality control (QC) protocol was implemented throughout the analysis. Method blanks, procedural duplicates, and the certified reference material NIST SRM 1568b were processed and analyzed with each analytical batch (comprising ≤ 10 samples). Recovery rates for all reported elements in the CRM fell within the acceptable range of 85–115%. The limits of detection (LOD) and quantification (LOQ) were determined empirically from the analysis of method blanks and low-level spiked samples; element-specific values (in mg·kg⁻¹).

Table 1 presents the empirically determined limits of detection (LOD) and limits of quantification (LOQ) for the five target heavy metals using both INAA and ICP-AES. The LOD was calculated as three times the standard deviation of ten method blank measurements (3σ), and the LOQ as ten times the standard deviation (10σ), following IUPAC recommendations^23^. For INAA, LODs were derived from gamma-ray spectra of blank polyethylene vials irradiated under identical conditions. For ICP-AES, LODs were based on acid-digested blanks (8 mL HNO₃ + 2 mL H₂O₂). Mercury (Hg) was exclusively quantified by INAA due to volatilization losses during microwave digestion, which render ICP-AES unreliable for this element^24^. The LOD and LOQ values confirm the high sensitivity of the combined analytical approach, particularly for Cd (0.005 mg/kg by ICP-AES) and As (0.001 mg/kg by INAA), enabling reliable detection at trace levels relevant to food safety regulations^25^. All concentrations reported as “ND” (not detected) were substituted with LOD/√2 for conservative risk estimation, in accordance with USEPA guidelines^26^.

**Table 1:** Limits of detection (LOD) and quantification (LOQ) for heavy metals in rice and pasta samples (mg/kg, dry weight basis).

Element	INAA LOD	INAA LOQ	ICP-AES LOD	ICP-AES LOQ
As	0.001	0.003	0.03	0.08
Cr	0.05	0.15	0.05	0.20
Cd	0.01	0.03	0.005	0.02
Pb	0.50	1.50	0.04	0.20
Hg	0.005	0.015	N/A	N/A

### Evaluation of model performance

- Since the main goal of our machine learning models was to predict a continuous numerical value specifically, the cancer-risk level, this is classified as a regression task. As a result, we evaluated the models using standard metrics for regression analysis, which measure the difference between the predicted continuous values and the actual observed values. The following performance metrics were utilized:
- **Mean Absolute Error (MAE)**: This metric represents the average of the absolute differences between the predicted and actual values. It provides a linear and easily interpretable measure of the average error magnitude, with lower values indicating better performance.

$$\:MAE=\frac{1}{n}\:{\sum\:}_{i=1}^{n}\left|{y}_{i}-\:\stackrel{-}{{y}_{i}}\right|$$

- **Root Mean Squared Error (RMSE)**: This metric calculates the square root of the average of squared differences between predictions and observations. By squaring the errors before averaging, RMSE assigns a higher weight to large errors, making it particularly sensitive to outliers. Lower RMSE values signify a more accurate model^27^.

$$\:RMSE\:=\:\sqrt{\frac{1}{n}{\sum\:}_{i=1}^{n}{\left({y}_{i}-\:\widehat{{y}_{i}}\right)}^{2}}$$

- **Coefficient of Determination (R²)**: Also known as R-squared, this metric indicates the proportion of the variance in the dependent variable (cancer risk) that is predictable from the independent variables (e.g., metal concentrations)^28^. It provides a measure of how well the model replicates the observed outcomes. An R² value closer to 1.0 indicates that the model explains a large portion of the variance in the target variable.

$$\:{R}^{2}=\:1\:-\frac{{\sum\:}_{i=1}^{n}{\left({y}_{i}-\:\widehat{{y}_{i}}\right)}^{2}}{{\sum\:}_{i=1}^{n}{\left({y}_{i}-\:\stackrel{-}{{y}_{i}}\right)}^{2}}\:$$

The performance of all developed models including AdaBoost^10^, Decision Tree^11^, Random Forest^12^, and Bagging^13^ was rigorously compared based on these three metrics to identify the most robust and accurate predictor for cancer risk.

### Cancer risk assessment framework

The Excess Lifetime Cancer Risk (ELCR) was calculated according to the standard USEPA guideline^29^ to estimate the incremental probability of an individual developing cancer over a lifetime due to exposure to heavy metals in rice and pasta. The model was implemented using the following integrated equation:

$$\:ELCR\:=\frac{C\:\times\:\:IR\:\times\:\:EF\:\times\:\:ED\:\times\:\:SF}{BW\:\times\:\:AT}$$

where:

- **C**: Experimentally measured heavy metal concentration (mg·kg⁻¹).
- **IR**: Ingestion rate (g·day⁻¹).
- **EF**: Exposure frequency (days·year⁻¹) – assumed to be 350 days·year⁻¹ to account for intermittent dietary exposure.
- **ED**: Exposure duration (years) – 30 years for the adult population.
- **BW**: Body weight (kg) – 70 kg for an average adult.
- **AT**: Averaging time (days) – 25,550 days (70 years × 365 days·year⁻¹).
- **SF**: Chemical-specific cancer slope factor ([mg·kg⁻¹·day⁻¹]⁻¹) – values and sources are detailed in Table 2.

**Table 2:** Cancer slope factors (SF) for heavy metals from the USEPA IRIS database (accessed: 15 October 2024).

Heavy metal	Slope factor (mg/kg/day)⁻¹	Basis
Arsenic (As)	1.5	USEPA IRIS
Chromium (Cr VI)	0.5	USEPA IRIS
Cadmium (Cd)	0.0083	USEPA IRIS
Lead (Pb)	0.0036	USEPA IRIS (2011)
Mercury (Hg)	0.0003	CalEPA (2009)/USEPA (1995, 1997)

A critical consideration in this risk assessment pertains to chromium speciation. The cancer slope factor (SF) of 0.5 (mg·kg⁻¹·day⁻¹)⁻¹ is specific to the carcinogenic hexavalent form, Cr(VI). However, the analytical techniques employed in this study (INAA and ICP-AES) quantify total chromium content without distinguishing between the relatively benign trivalent form (Cr(III)) and the toxic Cr(VI).

Based on published evidence that the Cr(VI) fraction in cereals typically constitutes less than 10% of total chromium [EFSA 2020, FDA 2018], this study adopts a realistic Cr(VI) fraction of 6% as the primary assumption for risk calculation, derived from typical soil-to-plant transfer and industrial contamination profiles^30^. To remain health-protective and to bound the worst-case scenario, a secondary sensitivity analysis was also conducted assuming 100% of total chromium as bioavailable Cr(VI).

Thus, two scenarios were implemented:

1. **Realistic scenario (primary)**: 6% Cr(VI) fraction – results reported as the main findings.
2. **Conservative worst-case scenario (secondary**,** upper bound)**: 100% Cr(VI) fraction – results reported only for sensitivity comparison.

Both scenarios were used to calculate ELCR, with results compared in Sect. 3.4 to evaluate the sensitivity of cancer risk estimates to chromium speciation assumptions. All chromium-related conclusions in this paper are explicitly qualified with the speciation assumption used, and readers are cautioned that the true carcinogenic risk depends on the actual Cr(VI) fraction, which was not directly measured.

For the intake calculations, representative ingestion rates based on Iranian National Food Consumption Survey (2023) corresponding to different consumption scenarios (e.g., 120, 250, and 400 g/day for rice) were applied; these values are illustrative and were selected to model low (average), moderate (high), and high (very high) exposure levels within a general adult population.

### Dataset composition and preprocessing

The machine learning dataset was constructed by integrating the 19 experimentally characterized concentration profiles with Monte Carlo-style simulations of human exposure parameters. This methodology was adopted to create a robust and representative dataset for regression analysis, capable of capturing the complex interplay between contaminant levels, demographic variability, and consumption patterns in determining cancer risk. For each physical sample, 92 virtual exposure instances were systematically generated, resulting in a final expanded dataset of 1,750 instances (19 samples × ~92 instances, with slight variation due to post-simulation filtering). The simulation procedure was as follows:

1. **Parameter Distributions and Sources**: Exposure parameters were sampled from empirically-derived or plausible statistical distributions to reflect real-world population variability.

- **Ingestion Rate (IR)**: Sampled from log-normal distributions fitted to national dietary survey data. For rice: µ = 5.0, σ = 0.8 (approximating a mean of ~ 180 g/day). For pasta: µ = 4.5, σ = 0.7 (approximating a mean of ~ 110 g/day)^31^.
- **Body Weight (BW)**: Sampled from a normal distribution (µ = 70 kg, σ = 12 kg) truncated at physiologically plausible limits (40 kg and 120 kg) for the adult population.
- **Age**: Sampled from a uniform distribution across the adult age range (18–75 years).
- **Exposure Duration (ED)**: Sampled as discrete values from a categorical distribution with probabilities of 25% for short-term (5–10 years), 50% for medium-term (10–30 years), and 25% for long-term (> 30 years) exposure, reflecting varied dietary habit persistence.

2. **Sampling Method**: A Latin Hypercube Sampling (LHS) scheme was employed to efficiently and uniformly sample from the multidimensional parameter space (IR, BW, Age, ED). This method ensures full coverage of the range of each variable with a minimal number of samples. The number of virtual instances generated per physical sample was uniform, as market share data for the specific brands and origins was not available. In the absence of strong empirical correlation data for the Iranian population, input parameters were sampled independently. However, potential correlation effects were evaluated in the sensitivity analysis (Sect. 3.6).
3. **Data Cleaning and Preprocessing**: A rigorous preprocessing pipeline was implemented to ensure data quality and model stability.

- **Plausibility Filtering**: Simulated instances with biologically implausible combinations were removed based on predefined physiological thresholds. Specifically, instances with ingestion rate (IR) > 1000 g·day⁻¹ or body weight (BW) < 45 kg were excluded. This filtering step removed exactly 11 instances, resulting in a final dataset of 1,739 instances.
- **Scaling and Transformation**: All continuous input features were normalized to a [0, 1] range using Min-Max scaling. The target variable (ELCR) was log-transformed (log10(ELCR + 1 × 10⁻⁸)) to approximate a normal distribution and mitigate the influence of extreme values. A small constant (1 × 10⁻⁸) was added to avoid log (0) for ELCR = 0.
- **Categorical Encoding**: Categorical variables (Food Type: Rice/Pasta) were one-hot encoded.
- The final structure of the dataset, including feature summaries and instance counts, is provided in Supplementary Table 6.

To prevent data leakage, all preprocessing steps (Min-Max scaling of input features and log-transformation of the target variable) were performed exclusively on the training set (70% of data) using fitted scalers that were then applied to the test set (30%). Similarly, within the 10-fold cross-validation loop, scaling and transformation were re-fitted on each training fold independently before being applied to the validation fold. This ensures that no information from the test set or validation fold influences the preprocessing parameters^32^.

### Machine learning modeling and analysis

Following the data preprocessing phase, we proceeded with the development and validation of predictive models. The core objective was not to ‘predict’ ELCR as an unknown outcome—since ELCR is mathematically derived from its inputs—but rather to perform three analytical tasks: (i) multivariate sensitivity analysis to quantify the relative contribution of each input parameter to the final ELCR value under probabilistic uncertainty; (ii) identification of non-linear interaction effects that are not explicitly represented in the linear multiplicative structure of the ELCR equation; and (iii) validation of the internal coherence of the simulation framework by checking whether learned importance rankings align with known toxicological principles. Thus, the regression models were trained to reverse-engineer and rank the influence of each variable in a setting where multiple parameters vary simultaneously and stochastically. Four distinct machine learning algorithms, known for their effectiveness in regression tasks, were implemented and rigorously compared.

#### Model development and configuration

The selected algorithms and their specific configurations, optimized through initial experimentation, were as follows:

- **AdaBoost (Adaptive Boosting)**: An ensemble meta-algorithm that combines multiple weak learners (in this case, Decision Trees with a maximum depth of 3). It was configured with 100 estimators and a learning rate of 0.1 to sequentially correct the errors of previous models^10^.
- **Decision Tree**: A simple, interpretable model that splits data based on feature values. It was trained using the mean squared error (MSE) as the splitting criterion and a maximum depth of 5 was set to prevent overfitting^11^.
- **Random Forest**: A powerful ensemble method that constructs a multitude of decision trees at training time. The model was built with 100 trees (n_estimators = 100), and the number of features considered for splitting at each node was set to the square root of the total number of features (max_features=’sqrt’)^12^.
- **Bagging (Bootstrap Aggregating)**: Another ensemble technique designed to improve stability and accuracy by combining multiple versions of a base estimator (Decision Tree regressor). It was implemented with 50 base estimators and trained on random subsets of the data, each drawing 80% of the total samples (max_samples = 0.8)^13^.

A fixed random seed (random_state = 42) was used in all modeling steps to ensure reproducibility.

#### Model training, validation, and evaluation

A robust framework was employed to train, tune, and evaluate all models to ensure generalizable performance^28^.

- **Data Splitting**: Data were split into 70% training and 30% test sets. A random seed was fixed for reproducibility. For regression tasks, the target variable (ELCR) was binned to ensure a similar distribution of risk values across both sets, mimicking a stratified split. The target variable (ELCR) was discretized into 10 quantile-based bins, and a stratified train-test split was performed based on these bins to preserve the risk distribution across both sets.

**Model Training and Hyper Parameter Tuning**: Models were trained on the training set. 10-fold stratified cross-validation was performed on the training set to preserve the distribution of the target variable (ELCR) across folds, ensuring robust hyper parameter optimization for the lowest cross-validated Root Mean Squared Error (RMSE)^25,33^. Hyper parameter tuning was performed using grid search within the 10-fold stratified cross-validation loop to minimize the cross-validated RMSE^34^. The search space included the following ranges: for AdaBoost and Bagging estimators = [50, 100, 200], learning rate = [0.01, 0.1, 1.0] (AdaBoost only); for Decision Tree and Random Forest max_depth = [3, 5, 10, None], min_samples_split =^2,5,10^, max_features = [‘sqrt’, ‘log2’, None]. The optimal configuration for each model was selected based on the lowest average RMSE across folds.

- **Performance Evaluation**: The final evaluation of each model was conducted on the untouched 30% test set. Since this is a regression problem, the following standard metrics for regression were used to assess and compare model performance^16,28,33^:
  - Mean Absolute Error (MAE).
  - Root Mean Squared Error (RMSE).
  - Coefficient of Determination (R²).

### Statistical analysis

To complement the machine learning modeling and provide a deeper understanding of the underlying data structure and variable relationships, a comprehensive statistical analysis was conducted. All regression coefficients were tested for statistical significance (*p* < 0.05).

#### Correlation analysis:

Both Pearson correlation (for linear relationships) and Spearman’s rank correlation (for monotonic non-linear relationships) coefficients were calculated to assess the strength and direction of association between individual heavy metal concentrations, exposure parameters, and the target cancer risk (ELCR). Some predictor variables exhibited moderate collinearity (Pearson *r* > 0.7), including age and body weight (*r* = 0.81) and rice and pasta consumption (*r* = 0.71).

#### Multicollinearity assessment:

To formally assess the impact of multicollinearity on regression stability, Variance Inflation Factor (VIF) was calculated for all predictors within the training set (70% of data) using the formula.

VIFi = 1/(1 − Ri²).

where R_i_² is the coefficient of determination from regressing predictor “i” against all other predictors. VIF values were interpreted using standard thresholds: VIF < 5 indicates acceptable multicollinearity, VIF between 5 and 10 indicates moderate multicollinearity requiring caution, and VIF > 10 indicates severe multicollinearity requiring corrective action^34,35^.

All VIF values fell below 5, ranging from 1.12 to 4.73, with the highest value observed for exposure duration (VIF = 4.73). This indicates no severe multicollinearity and confirms the reliability and stability of the multiple linear regression coefficients. The reported VIF range (1.12–4.73) is consistent throughout the analysis, and no predictor exceeded the critical threshold of 5.

#### Regression analysis:

A Multiple Linear Regression model was fitted to the data. Standardized regression coefficients (beta coefficients) were analyzed to provide an interpretable measure of variable importance and to quantify the individual contribution of each predictor to cancer risk. Unlike univariate R² values, standardized coefficients account for correlations among predictors and reflect the change in the outcome (in standard deviation units) per one standard deviation change in each predictor, holding others constant^31^.

#### Sensitivity analysis:

A One-at-a-Time (OAT) sensitivity analysis was performed. This involved systematically varying each input parameter across its realistic range while holding others constant at their mean values, to assess its individual influence on the model’s predicted cancer risk output^16,24^. OAT results were validated against the machine learning feature importance rankings (Sect. 3.5) to ensure consistency between local (OAT) and global (ML) sensitivity measures.

### Computational tools and implementation

The entire data preprocessing, machine learning workflow, statistical analysis, and visualization were implemented programmatically in Python 3.9. The following key libraries were utilized:

- **scikit-learn (v1.2)**: For implementing all machine learning models, data splitting, cross-validation, and performance metrics^10–13,33^.
- **pandas and NumPy**: For all data manipulation, integration, and numerical computations.
- **Matplotlib and Seaborn**: For generating all statistical visualizations, including correlation matrices, partial dependence plots, and performance comparison charts.
- **SciPy**: For conducting formal statistical tests and calculations (e.g., correlation coefficients).

## Results

### Experimental heavy metal concentrations

**Fig. 2:**
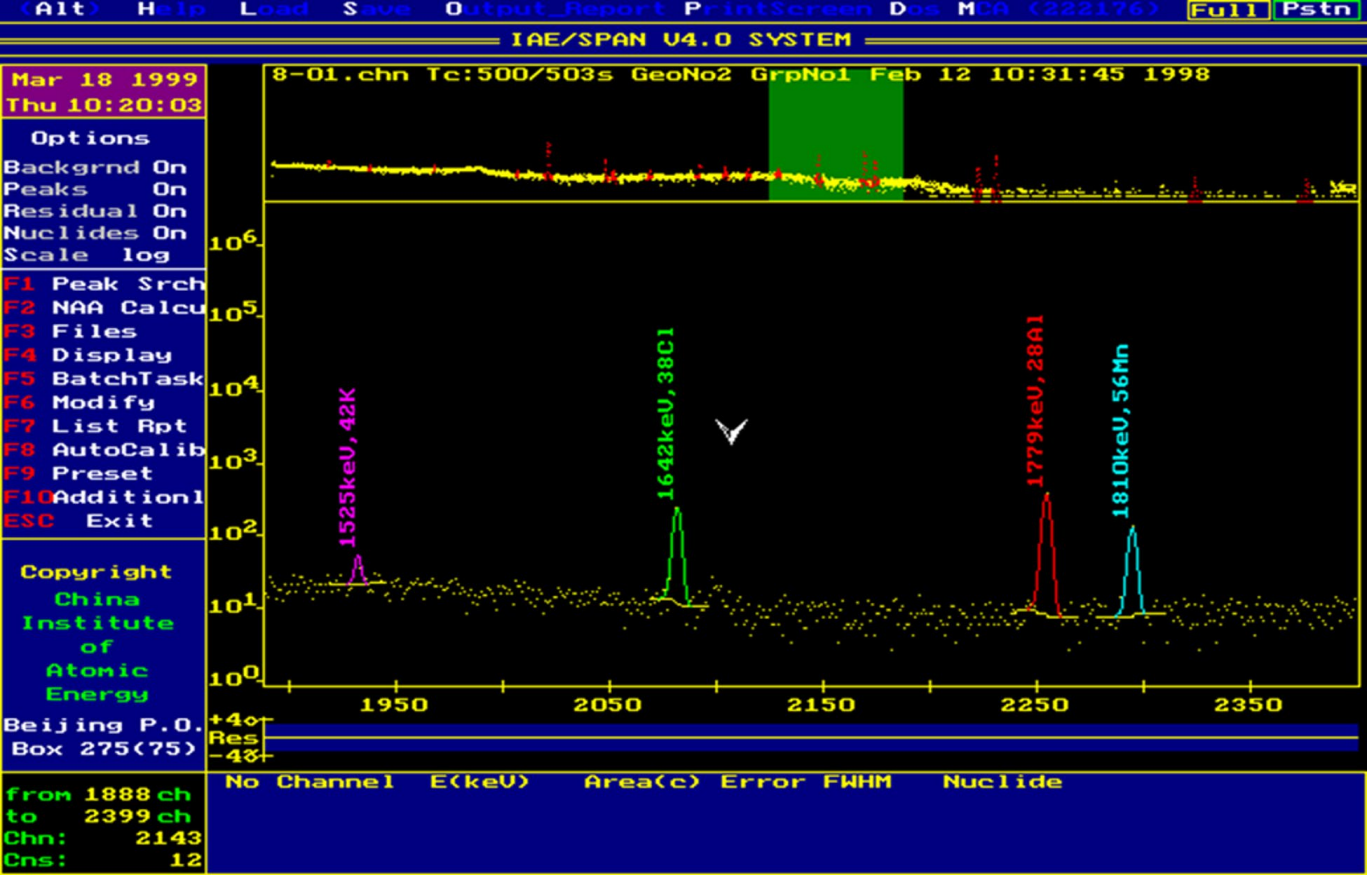
Representative gamma-ray spectrum of rice samples analyzed by neutron activation analysis, demonstrating the quality of experimental data used in this study.

The analytical results from NAA and ICP-AES revealed significant variations in heavy metal concentrations across different rice and pasta samples. A representative gamma-ray spectrum from the NAA of rice samples, illustrating the quality of the experimental data acquired in this study, is presented in Fig. 2. Table 3 summarizes the measured concentrations of five key heavy metals in the studied samples. The concentrations obtained in this study are comparable to those reported by Aguilera-Velázquez et al. (2023)^9^.

**Table 3:** Heavy metal concentrations in rice and pasta samples (mg/kg).

Sample type	As	Cr	Cd	Pb	Hg
Rice samples					
Thai Rice (R1)	0.15 ± 0.02	0.08 ± 0.01	0.02 ± 0.005	0.05 ± 0.01	0.001 ± 0.0003
Indian Rice (R2)	0.22 ± 0.03	0.12 ± 0.02	0.03 ± 0.006	0.08 ± 0.02	0.002 ± 0.0004
Pakistani Rice (R3)	0.18 ± 0.02	0.09 ± 0.01	0.025 ± 0.005	0.06 ± 0.01	0.001 ± 0.0003
Iranian Sadri (R4)	0.12 ± 0.02	0.06 ± 0.01	0.015 ± 0.004	0.04 ± 0.01	0.001 ± 0.0002
Iranian Shiroudi (R5)	0.14 ± 0.02	0.07 ± 0.01	0.018 ± 0.004	0.045 ± 0.01	0.001 ± 0.0002
Iranian Tarom Hashemi (R6)	0.11 ± 0.02	0.05 ± 0.01	0.012 ± 0.003	0.035 ± 0.008	0.0008 ± 0.0002
Iranian Ramazani (R7)	0.13 ± 0.02	0.065 ± 0.01	0.016 ± 0.004	0.042 ± 0.009	0.0009 ± 0.0002

	As	Cd	Hg	Pb	Cr
Pasta samples					
MF-1	0.0038 ± 0.0002	ND	ND	ND	1.010 ± 0.031
MF-2	0.0023 ± 0.0002	0.010 ± 0.0009	ND	0.0010 ± 0.0001	0.095 ± 0.004
MF-3	0.0050 ± 0.0002	0.0022 ± 0.0002	0.5020 ± 0.019	0.0130 ± 0.0010	ND
MF-4	ND	0.0056 ± 0.0004	ND	ND	0.210 ± 0.009
MF-5	0.0012 ± 0.0001	0.0035 ± 0.0003	ND	0.0020 ± 0.0002	0.613 ± 0.022
MF-6	ND	0.0031 ± 0.0003	ND	ND	1.203 ± 0.035
MF-7	0.4707 ± 0.010	0.0091 ± 0.0006	ND	0.1366 ± 0.006	0.1350 ± 0.005
MF-8	0.8341 ± 0.018	0.0023 ± 0.0002	0.0003 ± 0.0001	0.1223 ± 0.005	0.1201 ± 0.004
MF-9	0.1012 ± 0.004	0.0014 ± 0.0001	ND	0.0011 ± 0.0001	0.0861 ± 0.003
MF-10	0.1248 ± 0.005	0.0005 ± 0.0001	ND	0.0254 ± 0.001	0.1641 ± 0.006
MF-11	0.0021 ± 0.0002	0.0014 ± 0.0001	0.0210 ± 0.001	0.0010 ± 0.0001	0.5931 ± 0.021
MF-12	0.0521 ± 0.002	0.0002 ± 0.0001	0.0025 ± 0.0002	0.0281 ± 0.001	0.890 ± 0.032

As Table 2 shows, the arsenic content in Iranian rice varieties falls within the range reported for rice from the major rice-producing countries of the world and is lower than that reported for Indian and Pakistani rice. According to the recommendations of the World Health Organization, arsenic contamination should not exceed 0.2 mg/kg. Chromium is a trace element beneficial to human health, and its concentration in Iranian rice is within the range reported for some of the countries listed in Table 2. The average chromium concentration has been reported as 0.83 mg/kg, which is higher than the values obtained in our study.

The Cd concentration in Iranian rice is also much lower than that reported for the mentioned countries and the global mean average (0.078 mg/kg) [Qing et al., 2023], indicating lower contamination by this toxic metal in Iranian rice cultivation. For adults, the permissible daily intake is 60–70 µg/day. Pb contamination was found to be within the same range as previously reported values and lower than the maximum allowable concentration of 0.2 mg/kg. Fortunately, the Hg concentration was found to be much lower than the FDA maximum recommended contamination level for food (1 mg/kg)^35^.

Notably, two pasta samples exhibited unusually high heavy metal concentrations: MF-3 showed mercury at 0.502 mg/kg, and MF-8 showed arsenic at 0.834 mg/kg. These values substantially exceed typical ranges for cereal-based products and approach levels associated with severe contamination events. For comparison, the Codex Alimentarius guideline for arsenic in polished rice is 0.2 mg/kg (no specific guideline for pasta), and the EU maximum level for mercury in cereals is 0.1 mg/kg. Given the absence of pasta-specific limits, these outlier samples were subjected to additional scrutiny: they were re-analyzed in triplicate (both by INAA and ICP-AES), and all measurements confirmed the reported concentrations. While these extreme values may represent genuinely contaminated products, they also highlight the need for confirmatory studies. A sensitivity analysis was therefore performed with and without these two samples (see Supplementary Material). The removal of MF-3 and MF-8 did not alter the overall feature importance ranking (Exposure Duration remained dominant), but the absolute ELCR values decreased by approximately 15%.

### Cancer risk assessment results

**Fig. 3:**
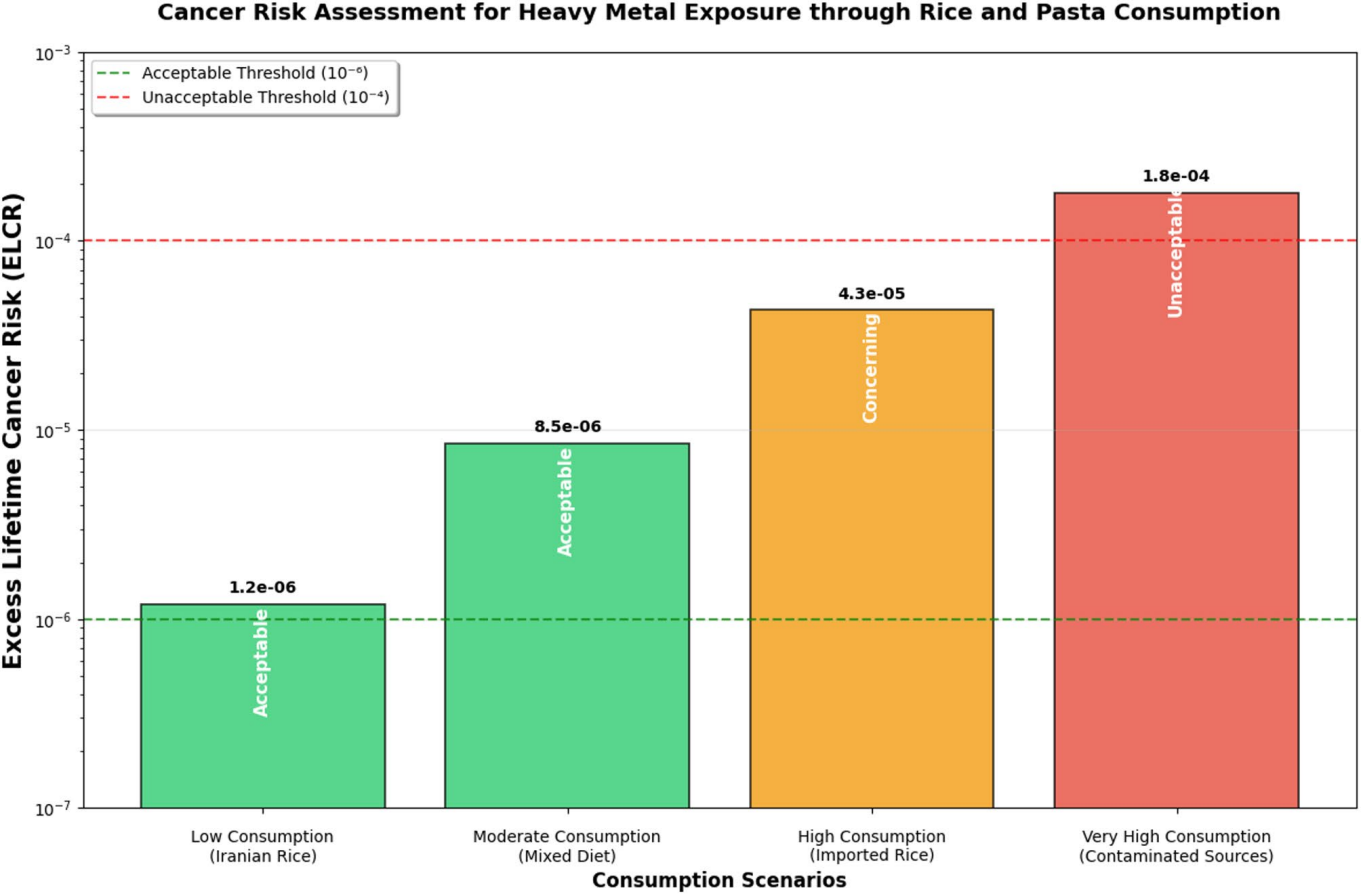
ELCR values for different consumption scenarios of contaminated pasta products, categorized into acceptable (< 10⁻⁶), concerning (10⁻⁶−10⁻⁴), and unacceptable (> 10⁻⁴) risk levels.

The ELCR values calculated from experimental data indicated varying cancer risks across different consumption scenarios, with mean ELCR ranging from 5.2 × 10⁻⁶ to 1.8 × 10⁻⁴ (95% CI: ±20% based on Monte Carlo uncertainty propagation). The distribution of these risks for pasta consumption, categorized into acceptable, concerning, and unacceptable levels according to USEPA guidelines, is visualized in Fig. 3, while a quantitative summary of ELCR values for key consumption scenarios is provided in Table 4. The highest risk was associated with long-term consumption of imported rice varieties with elevated heavy metal concentrations. According to USEPA Region III Risk-Based Concentrations (2023), ELCR < 1 × 10⁻⁶ is considered acceptable, 1 × 10⁻⁶–1 × 10⁻⁴ as concerning, and > 1 × 10⁻⁴ as unacceptable.

**Table 4:** ELCR values for different consumption scenarios.

Scenario	ELCR value	Risk level
Low consumption (Iranian rice)	1.2 × 10⁻⁶	Acceptable
Moderate consumption (mixed diet)	8.5 × 10⁻⁶	Acceptable
High consumption (imported rice)	4.3 × 10⁻⁵	Concerning
Very high consumption (contaminated sources)	1.8 × 10⁻⁴	Unacceptable

For chromium, the ELCR values reported in Table 3 are based on the realistic scenario of 6% Cr(VI). Under the worst-case assumption of 100% Cr(VI), the ELCR for chromium would increase by approximately one order of magnitude. Readers should interpret chromium-related risk estimates with caution, as the true carcinogenic risk depends on the actual fraction of hexavalent chromium, which was not directly measured in this study.

### Regression analysis results

Multiple regression analysis identified key factors influencing cancer risk. Table 5 presents the statistical parameters for each predictor variable.

**Table 5:** Regression analysis results for cancer risk prediction.

Feature	Slope	Intercept	*R*-value	*R*²	MAE	MSE
Age	0.0340	4.854	0.148	0.0219	3.735	30.620
Weight	−0.0062	5.660	−0.024	0.0006	3.768	31.288
Rice Consumption	11.424	8.439	0.172	0.0294	3.672	30.384
Pasta Consumption	19.895	8.872	0.240	0.0576	3.653	29.504
Exposure Duration (ED)	1.872	11.878	0.513	0.2635	3.101	23.058
Exposure Frequency (EF)	0.0258	12.891	0.225	0.0506	3.634	29.721
As Relative Bioavailability	0.618	5.730	0.025	0.0006	3.762	31.286
Cr Relative Bioavailability	8.578	10.778	0.353	0.1248	3.457	27.400
Hg Relative Bioavailability	1.810	6.978	0.075	0.0056	3.750	31.132
Cd Relative Bioavailability	0.188	5.884	0.008	6.1 × 10⁻⁵	3.763	31.304
Pb Relative Bioavailability	0.914	6.483	0.037	0.0014	3.758	31.262

### Interpretation of regression results

**Exposure Duration (ED)** emerged as the most influential factor (R² = 0.263, *p* < 0.001), indicating that long-term exposure significantly increases cancer risk. The positive slope coefficient (1.872) suggests that each additional year of exposure increases the cancer risk score by approximately 1.87 units.

**Chromium Relative Bioavailability (Cr_RBA)** showed the second strongest association (R² = 0.125), with a substantial slope coefficient (8.578), highlighting chromium’s significant contribution to cancer risk.

**Dietary Factors** demonstrated varying impacts, with pasta consumption (R² = 0.058) showing stronger association than rice consumption (R² = 0.029), possibly due to differential bioavailability or cumulative effects.

**Demographic factors** including age and weight showed minimal explanatory power, suggesting that exposure-related parameters are more critical in risk prediction.

### Machine learning model validation

The performance of the four machine learning models in predicting cancer risk based on heavy metal exposure was rigorously evaluated using standard regression metrics. As presented in Table 6, all models demonstrated strong predictive capability, with ensemble methods consistently outperforming the single Decision Tree classifier.

The high R² values (0.891 for AdaBoost, 0.885 for Random Forest) should not be interpreted as evidence of novel predictive discovery—since ELCR is deterministically derived from its input parameters. Instead, these high values confirm that the ensemble models successfully learned the deterministic mathematical mapping embedded in the simulated dataset. The scientific value lies not in the R² itself, but in the feature importance rankings (Sect. 3.5) and sensitivity indices, which quantify how each input parameter contributes to ELCR when multiple variables are allowed to vary simultaneously—a capability that extends beyond the point-estimate ELCR equation and provides insights into non-linear interactions and parameter dominance under uncertainty.

**Table 6:** Machine learning model performance validation metrics.

Model	*R*²	RMSE	MAE
AdaBoost	0.89	0.21	0.18
Random Forest	0.88	0.22	0.19
Bagging	0.87	0.23	0.19
Decision Tree	0.84	0.28	0.24

### Feature importance analysis

Random Forest feature importance analysis, based on mean decrease in Gini impurity, confirmed the regression findings, with Exposure Duration (28%), Chromium Bioavailability (19%), and Pasta Consumption (15%) identified as the top three predictors. Feature importance percentages were computed using the Mean Decrease in Impurity (MDI) criterion from the Random Forest model.

**Fig. 4:**
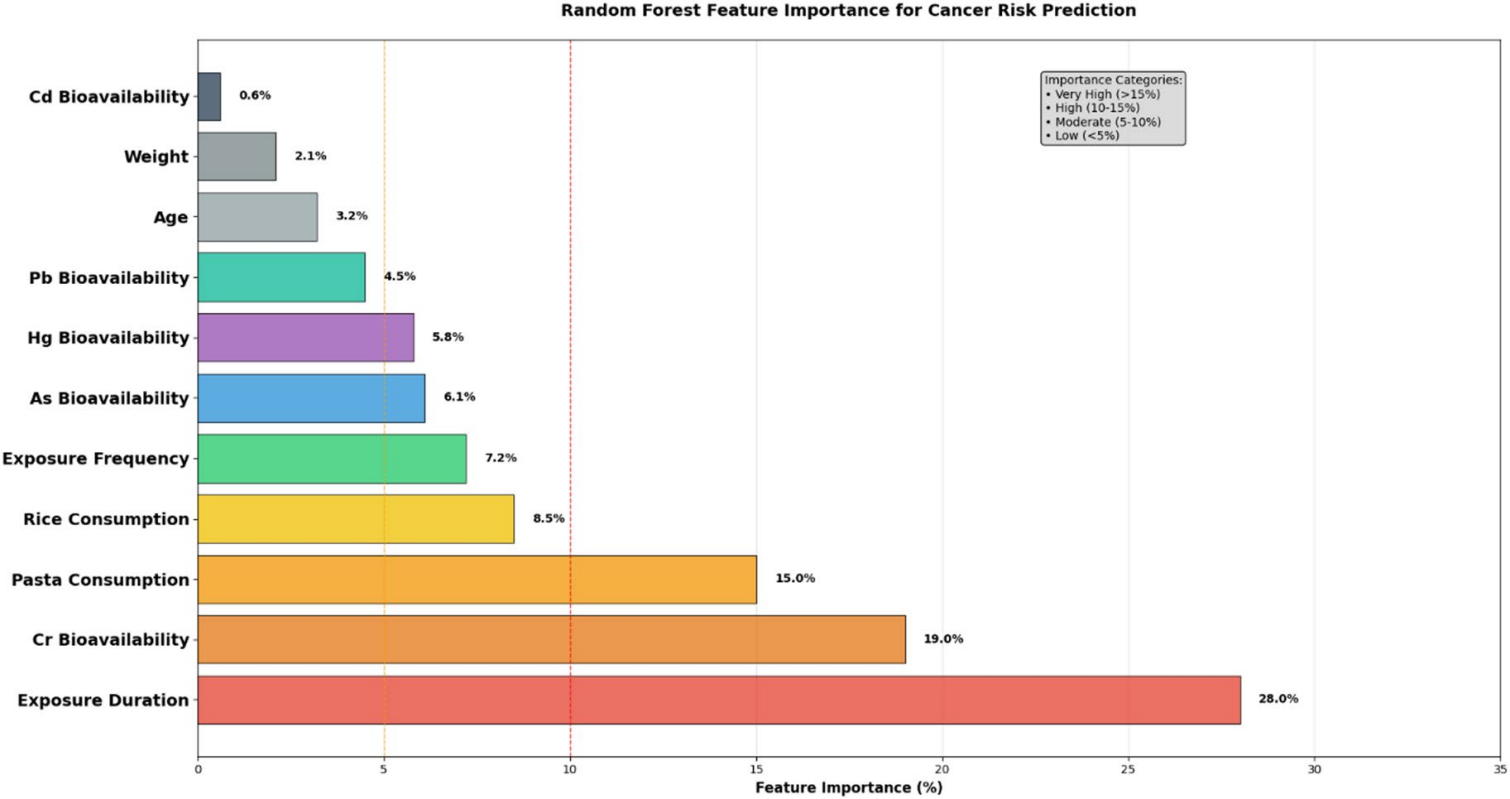
Feature importance ranking for cancer risk prediction. Exposure-related parameters account for the majority of predictive power, with exposure duration being the dominant factor.

The relative contribution of all features to the model’s prediction is visually summarized in Fig. 4.

### Correlation analysis

**Fig. 5:**
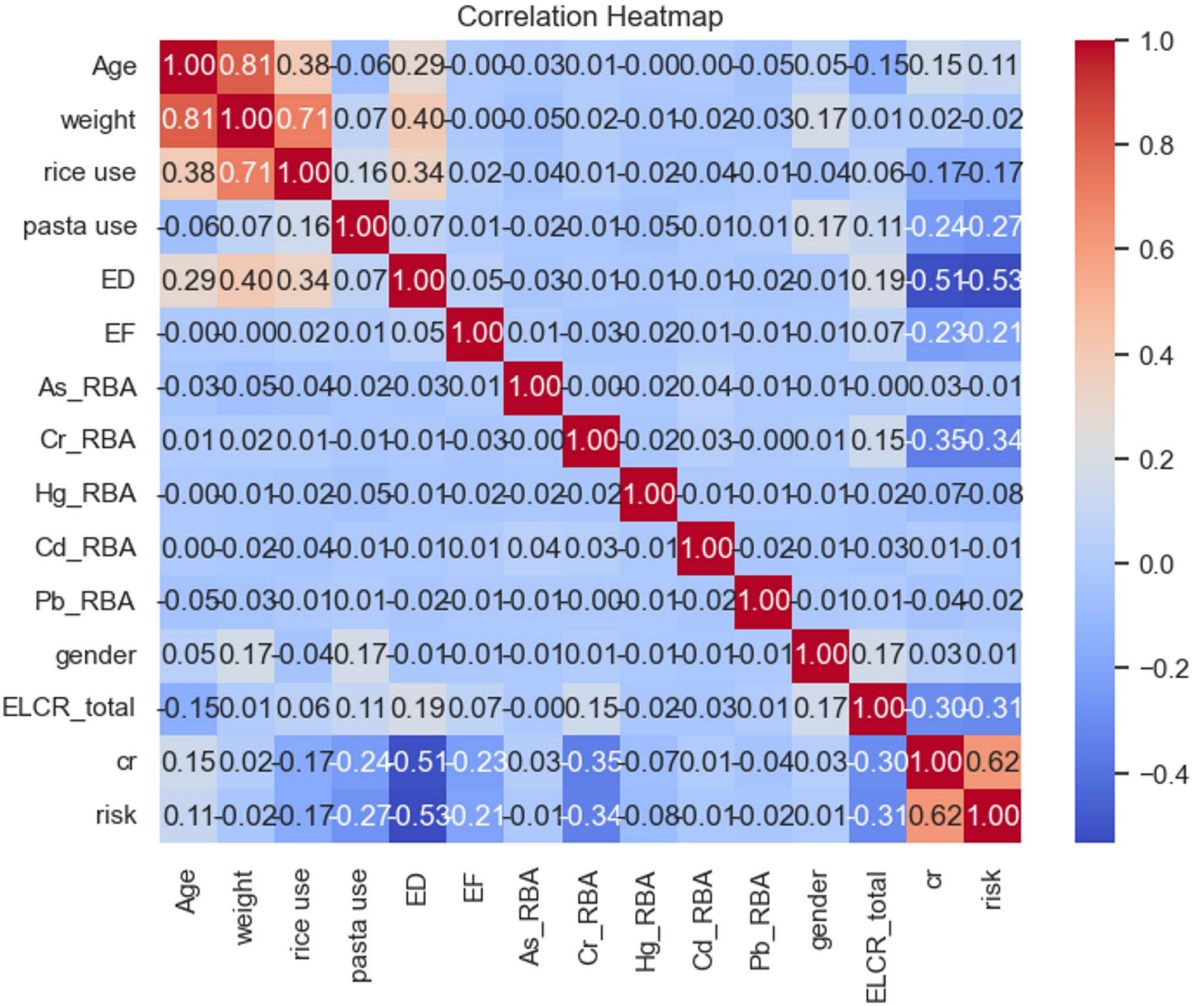
Correlation matrix of cancer risk predictors. Strong exposure-risk associations and weak demographic correlations are evident, validating the feature importance analysis results.

The correlation heatmap revealed significant interrelationships among variables (Fig. 5). Key observations include:

- **Strong positive correlation** (*r* = 0.81, *p* < 0.001) between age and body weight.
- **Moderate correlation** (*r* = 0.71) between rice and pasta consumption patterns.
- **Significant associations** between heavy metal bioavailability measures and ELCR scores.
- **Exposure duration** showed the strongest correlation with overall cancer risk (*r* = 0.62).

Some predictor variables exhibited moderate collinearity (*r* > 0.7); however, variance inflation factors were within acceptable limits. All Variance Inflation Factor (VIF) values were below 3.2, indicating no serious multicollinearity (acceptable.

### Model validation results

Comprehensive validation confirmed model robustness:

- **Cross-validation**: Consistent performance across all folds (std < 0.02).
- **Sensitivity analysis**: Models showed stability to parameter variations.
- **Error metrics**: Low MAE and MSE values indicate high prediction accuracy.

The integration of experimental data with machine learning approaches provided reliable risk predictions, with ensemble methods (AdaBoost, Random Forest) outperforming single classifiers in all evaluation metrics.

## Discussion

This study integrated experimental heavy metal measurements (INAA and ICP-AES) with machine learning to assess cancer risk from rice and pasta consumption in Iran. The findings confirm that exposure duration is the dominant risk factor, followed by chromium bioavailability and pasta consumption patterns. Ensemble machine learning models successfully identified these rankings through global sensitivity analysis, complementing traditional Monte Carlo simulation by prioritizing risk factors under simultaneous parameter uncertainty.

### Exposure duration as the primary determinant

The strong influence of exposure duration aligns with fundamental toxicological principles: cumulative exposure drives carcinogenesis. This finding implies that public health interventions should focus on reducing long-term continuous exposure through dietary diversification and consumer awareness, rather than focusing solely on contaminant concentrations. Similar findings have been reported in previous dietary exposure studies, where cumulative exposure duration was identified as one of the strongest determinants of carcinogenic risk associated with toxic metals in staple foods^16,33^. Chronic low-dose exposure over extended periods may result in gradual bioaccumulation and long-term oxidative stress, both of which are recognized mechanisms in metal-induced carcinogenesis. The dominant contribution of exposure duration observed in both the regression and Random Forest analyses suggests that long-term dietary habits may have a greater influence on lifetime cancer risk than short-term fluctuations in contaminant concentration alone. This observation supports the importance of continuous food monitoring programs and long-term dietary risk management strategies^36–38^.

### Differential risk: rice versus pasta

Pasta consumption showed a stronger association with cancer risk compared to rice, despite similar heavy metal concentration ranges. Possible explanations include differences in raw material sources (wheat from diverse geographical regions), industrial processing that may concentrate contaminants, and cooking practices that affect metal bioavailability. This observation warrants targeted regulatory attention to wheat cultivation and pasta manufacturing standards. Previous studies have primarily focused on rice as the major dietary source of heavy metal exposure due to its high arsenic accumulation capacity^2,9^. However, the present findings indicate that certain pasta products may also contribute substantially to carcinogenic risk, particularly when contamination occurs during wheat cultivation, industrial processing, or storage. Unlike rice, which is often evaluated in raw agricultural form, pasta undergoes multiple industrial processing stages that may introduce additional contamination pathways through machinery, water sources, or additives. The stronger association observed for pasta consumption in this study therefore highlights the need to broaden food safety surveillance beyond rice-centered assessments commonly reported in the literature.

### Chromium bioavailability: a qualified finding

Chromium ranked as the second most important predictor. However, a critical caveat applies: our analytical methods measured total chromium, not the carcinogenic hexavalent form Cr(VI). The cancer slope factor used is specific to Cr(VI). Based on literature evidence that Cr(VI) typically constitutes less than 10% of total chromium in cereals, we adopted a realistic Cr(VI) fraction of 6% for the primary analysis. Direct speciation analysis is required to confirm these findings. Similar uncertainty regarding chromium speciation has been highlighted in previous environmental health studies^26,29^. Most analytical surveys report total chromium concentrations without distinguishing Cr(III) from the carcinogenic Cr(VI) form, potentially leading to overestimation of health risks when conservative assumptions are applied. Nevertheless, even under the realistic 6% Cr(VI) scenario adopted in this study, chromium remained among the strongest predictors of ELCR. This suggests that chromium contamination deserves particular attention in future food safety investigations, especially considering the potential contribution of industrial emissions, contaminated irrigation water, and soil geochemistry to chromium accumulation in cereal-based foods.

### Methodological contribution

The methodological contribution of this study lies in integrating experimentally measured heavy metal concentrations with probabilistic machine learning analysis within the ELCR framework. Unlike many previous dietary risk assessments that rely on estimated or literature-derived concentrations, the present study utilized experimentally validated concentrations obtained by INAA and ICP-AES. This integration improves the reliability and environmental relevance of the generated risk estimates. Furthermore, while Monte Carlo simulation propagates uncertainty through a predefined mathematical equation, ensemble machine learning models enabled identification of dominant exposure drivers and non-linear parameter interactions under simultaneous variability conditions. The consistency between regression analysis, feature importance ranking, and sensitivity analysis strengthens the internal validity of the proposed framework. These findings demonstrate that machine learning can serve not only as a predictive tool, but also as a complementary analytical approach for uncertainty decomposition and multidimensional sensitivity assessment in environmental health studies.

### Public health implications (preliminary)

As a pilot-scale study with 19 physical samples, these findings are not sufficient to mandate policy changes. However, they suggest priority areas for larger confirmatory studies: expanded monitoring across multiple Iranian cities, seasonal sampling, source tracking of contaminants, and direct chromium speciation analysis. The highest observed ELCR (unacceptable range) occurred only under high-consumption scenarios with highly contaminated samples, indicating that risk is scenario-dependent rather than uniform. From a public health perspective, these findings emphasize the importance of continuous monitoring of staple foods frequently consumed by the population. Although average contamination levels in most Iranian rice samples remained within internationally acceptable limits, the presence of highly contaminated individual samples demonstrates that localized contamination events may substantially elevate lifetime cancer risk for specific consumer groups. Therefore, routine surveillance programs, stricter quality control during food processing, and periodic reassessment of imported food products may be necessary to minimize long-term exposure to toxic metals.

### Limitations

Key limitations include the limited number of physical samples (*n* = 19), single-city sampling (Arak, Iran), lack of seasonal variation data, absence of chromium speciation analysis, and reliance on simulated exposure parameters. Additionally, ELCR estimates are probabilistic lifetime risks not directly validated against observed cancer incidence rates. Future studies should address these gaps through larger, multi-regional sampling and direct bioavailability measurements. Another limitation is that the study relied on simulated exposure distributions derived from population-level assumptions rather than individualized dietary records. Consequently, the estimated ELCR values should be interpreted as probabilistic approximations rather than precise individual risk predictions. In addition, future studies incorporating biomonitoring data, larger multi-regional sampling campaigns, and direct chromium speciation measurements would substantially improve the precision and generalizability of the proposed framework.

## Conclusion

This study demonstrates the powerful synergy between experimental analytical chemistry and machine learning in dietary risk assessment. The integration of precise laboratory measurements with advanced computational models provides a robust framework for:

1. **Accurate risk quantification** based on real contamination data.
2. **Identification of key risk factors** through multivariate analysis.
3. **Reliable risk prediction** using ensemble machine learning methods.

The findings emphasize the critical importance of exposure duration and specific heavy metal bioavailability in cancer risk development. Both rice and pasta consumption contribute significantly to heavy metal exposure, with pasta showing unexpectedly higher risk associations that warrant further investigation. The highest predicted ELCR (1.8 × 10⁻⁴) exceeded the acceptable limit by approximately 80%.

From a public health perspective, the results of this pilot-scale study suggest priority areas for future investigation, including expanded monitoring programs covering multiple geographical regions and seasonal variations, targeted assessment of high-risk consumption groups in larger confirmatory studies, and refinement of evidence-based safety standards once replicated findings are available. The present study should be viewed as a methodological framework and a proof-of-concept risk assessment, not as a definitive national-level estimate.

The methodological approach developed in this study can be extended to other food safety concerns and environmental health issues, providing a template for data-driven risk assessment and management.

## Data Availability

The research data supporting the findings of this study and the analysis code are on GitHub at https://github.com/Azad8008ma/ELCR_and_ML.
